# Supplementary material for: Cas9/gRNA-Mediated Mutations in PtrFLA40 and PtrFLA45 Reveal Redundant Roles in Modulating Wood Cell Size and SCW Synthesis in Poplar
Source: Int J Mol Sci. 2022 Dec 27;24(1):427. doi: 10.3390/ijms24010427 (PMC9820481; doi:10.3390/ijms24010427)
Supplement: Supplementary file 1 [file ijms-24-00427-s001.zip › Figures S1-S4.pdf]

Supplementary Figures

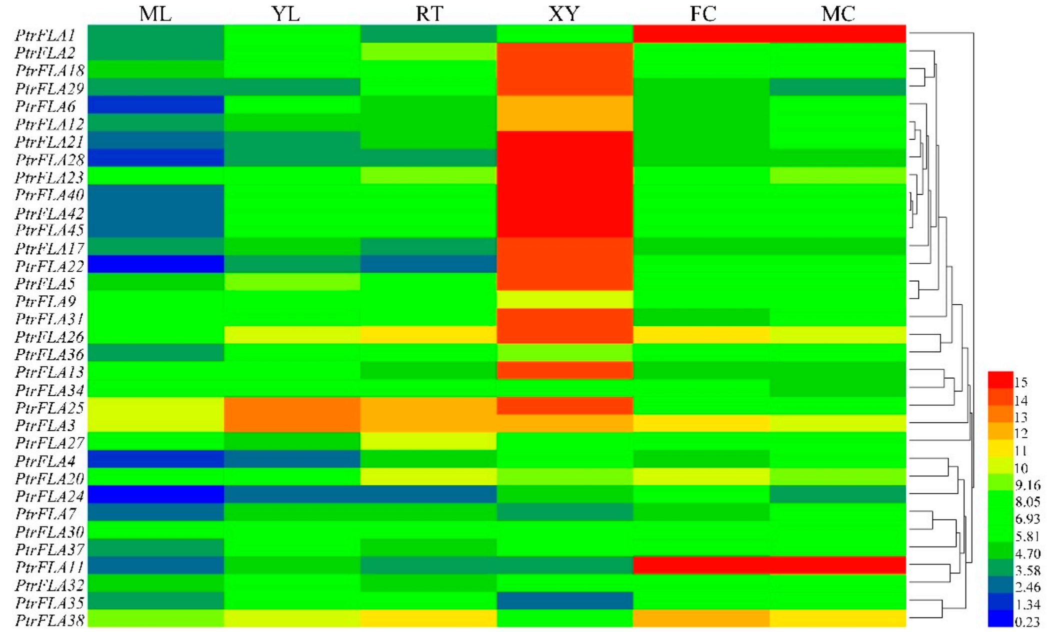

Figure S1. Hierarchical clustering of expression profiles of *PtrFLA* genes in different tissues. The microarray data were downloaded from the Poplar eFP browser. Color scale at the right of the dendrogram represents log2 expression values. ML, mature leaf; YL, young leaf; RT, root; XY, xylem; FC, female catkin; MC, male catkin.

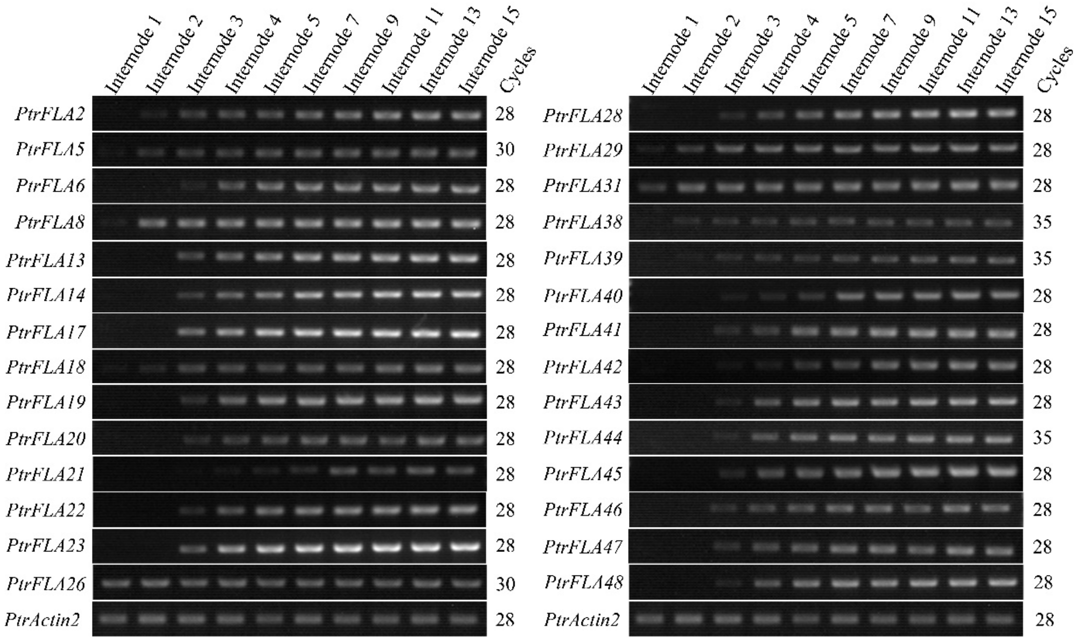

Figure S2. Gene expression of 28 *PtrFLAs* from group A in different stem internodes by RT-PCR analysis. Stem internodes included 1st to 5th, 7th, 9th, 11th and 12th internodes. The expression of *PtActin2* was used as an internal control.

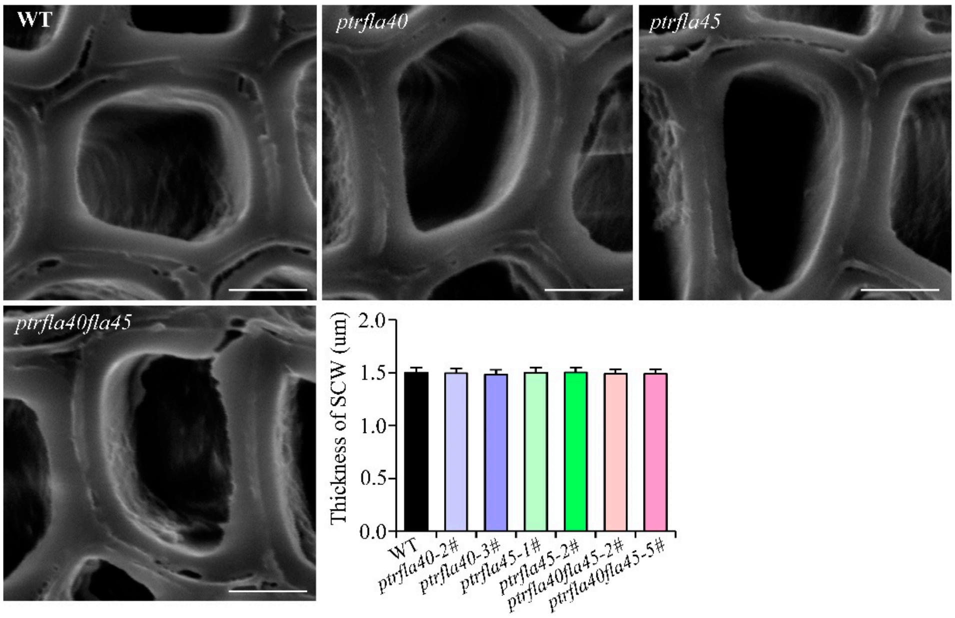

Figure S3. Wall thickness of secondary xylem mature fibers in 12th stem internodes of 4-month-old wild-type (WT), *ptrfla40*, *ptrfla45* and *ptrfla40fla45* mutants. Data are means ± SD (n=50). Bars: 5μm.

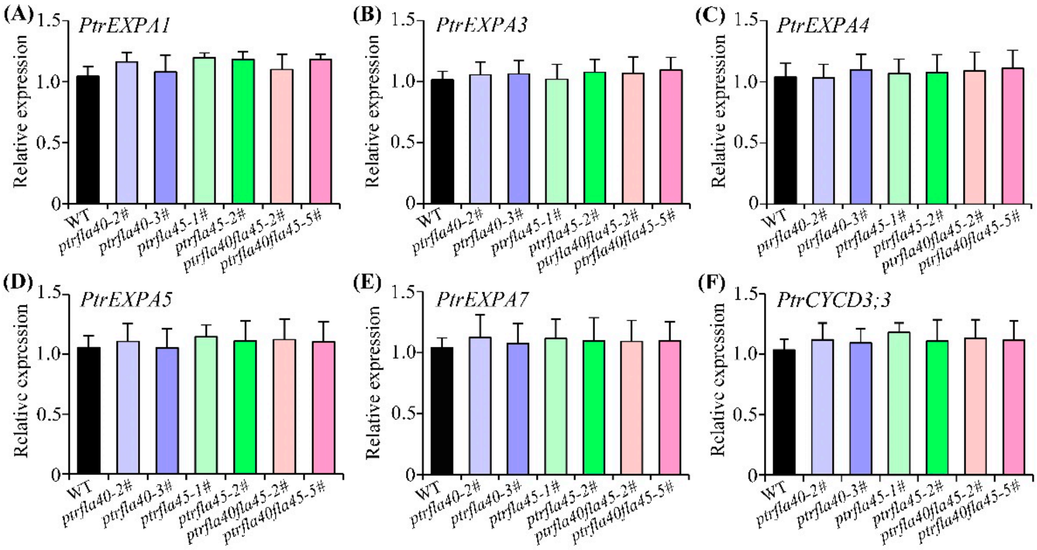

Figure S4. Transcription levels of five expansin genes and one cambial activity marker gene *PtrCYCD3;3* in wild-type (WT), *ptrfla40*, *ptrfla45* and *ptrfla40fla45* mutants by RT-qPCR analysis. The *PtActin2* was used as an internal control. Data are means ± SD (n=3).
