# Supplementary material for: Cas9/gRNA-Mediated Mutations in PtrFLA40 and PtrFLA45 Reveal Redundant Roles in Modulating Wood Cell Size and SCW Synthesis in Poplar
Source: Int J Mol Sci. 2022 Dec 27;24(1):427. doi: 10.3390/ijms24010427 (PMC9820481; doi:10.3390/ijms24010427)
Supplement: Supplementary file 1 [file ijms-24-00427-s001.zip › Table S3.pdf]

Table S3. All primers used in this study.

Usage	Primer Name	Sequence (5’-3’)
Primers for CRISPR/Cas9 vectors construction		
Cas9/gRNA-PtrFLA40	DT1-BsF/ PtrFLA40	ATATATGGTCTCGATTGATATATGTAGGGTTGTGTCGTT
	DT1-F0/ PtrFLA40	TGATATATGTAGGGTTGTGTCGTTTTAGAGCTAGAAATAGC
	DT2-R0/ PtrFLA40	AACAAGACGAAAACCGCTTATTCAATCTCTTAGTCGACTCTAC
	DT2-BsR/ PtrFLA40	ATTATTGGTCTCTAAACAAGACGAAAACCGCTTATTC
Cas9/gRNA-PtrFLA45	DT1-BsF/ PtrFLA45	ATATATGGTCTCGATTGTCACAAAGTTATACAAACTGTT
	DT1-F0/ PtrFLA45	TGTCACAAAGTTATACAAACTGTTTTAGAGCTAGAAATAGC
	DT2-R0/ PtrFLA45	AACTGGTTTAACCATCTTTGCACAATCTCTTAGTCGACTCTAC
	DT2-BsR/ PtrFLA45	ATTATTGGTCTCTAAACTGGTTTAACCATCTTTGCAC
Cas9/gRNA-PtrFLA40FLA45	DT1-BsF/ PtrFLA40FLA45	ATATATGGTCTCGATTGCGCTAAATGACTCAAGCCCGTT
	DT1-F0/ PtrFLA40FLA45	TGCGCTAAATGACTCAAGCCCGTTTTAGAGCTAGAAATAGC
	DT2-R0/ PtrFLA40FLA45	AACTTGTCCCAATACCTTTGCCCAATCTCTTAGTCGACTCTAC
	DT2-BsR/ PtrFLA40FLA45	ATTATTGGTCTCTAAACTTGTCCCAATACCTTTGCCC
Primers used to identify Cas9-gRNAs targeted sites		
gPtrFLA40-identify	Identify PtrAP40-F	TAACTTGATCCTCTGCTCACAAC
	Identify PtrAP40-R	GTCTGTGTATACGGTGCCAGAT
gPtrFLA45-identify	Identify PtrAP45-F	TATTGCACCTAACTATATAACCTC
	Identify PtrAP45-R	GGCTAGTTGGTTGTCTGTGTATA
Primers used to identify transgenic plants		

Note: Underlined letters come from PAM.

zCas	zCas-F	TGAGAACATCGTCATTGAGATGG
	zCas-R	CAGCTTGTCATTCTCATCGTACT
Hyg	Hyg-F	GAGCTTGTCGATCGACAGAT
	Hyg-R	CATATGCGCGATTGCTGATC
M13	M13F	TGTAAAACGACGGCCAGT
	M13R	CAGGAAACAGCTATGACC
Primers of PtrFLAs used for RT-qPCR and RT-PCR		
PtrFLA2/Potri.001G320800	PtrFLA2-F	GGCTGGCCACTTCAAGACCT
	PtrFLA2-R	GCAGGTGCCGGTGGTGGAGG
PtrFLA5/Potri.002G223300	PtrFLA5-F	CCTAACTCAAGACCAGGTGAA
	PtrFLA5-R	CCTGGAGCGCTCCCATCTCC
PtrFLA6/Potri.004G210600	PtrFLA6-F	GGCTGGCCACTTCACTGTC
	PtrFLA6-R	ATCAGTGTATACGGTGCCGG
PtrFLA8/Potri.006G129200	PtrFLA8-F	GCACCACCTGACAACGCCTT
	PtrFLA8-R	GGTGCTGGTGCTGCCGCTGT
PtrFLA13/Potri.009G012100	PtrFLA13-F	CTGGCTCCTCTTCCTCCATG
	PtrFLA13-R	AAGCGGGAACTTCCGGTCTG
PtrFLA14/ Potri.009G012200	PtrFLA14-F	GCAGACTCCTCACAGGCACC
	PtrFLA14-R	GTCAGTGTATACAGTGCCTG
PtrFLA17/Potri.012G015000	PtrFLA17-F	TCCCAGACACCTACAGCAGC
	PtrFLA17-R	CCTGATAGATAGCAAGCTGGC
PtrFLA18/Potri.012G127900	PtrFLA18-F	CCACCTGGTCCAACCAATGT
	PtrFLA18-R	CACTGAGTTACCTGTGGTGG
PtrFLA19/ Potri.013G014200	PtrFLA19-F	CACAGGCAGCTCAACCACAC
	PtrFLA19-R	CTGGTATTGGTAAGTCCTG

PtrFLA20/ Potri.013G120600	PtrFLA20-F	GCCACACTCATTCGCCTTCT
	PtrFLA20-R	CAGACACCATCTTGGCCTGA
PtrFLA21/Potri.013G151300	PtrFLA21-F	ATGCTTCTTCTTTCCCTGTG
	PtrFLA21-R	GATGGTTATACCAGTGTTGG
PtrFLA22/Potri.013G151400	PtrFLA22-F	GTTTCTAGTAGAATAGCCTGG
	PtrFLA22-R	GTCTGAAACTGGGAAGTGGAT
PtrFLA23/Potri.013G151500	PtrFLA23-F	GCACAGGCACCAGCAGCAGT
	PtrFLA23-R	CTGGTGCAGGTGCTACCGGT
PtrFLA26/Potri.014G162900	PtrFLA26-F	CACCATCACCTCCTTACGTT
	PtrFLA26-R	GCTCACTTGGCTAAGGTTCT
PtrFLA28/Potri.015G013300	PtrFLA28-F	CCCAGCCACCAAATGCAGCA
	PtrFLA28-R	GGCCGAAAAGGCACCATCAG
PtrFLA29/Potri.015G129400	PtrFLA29-F	GCACCACCCGGTCCAACCAA
	PtrFLA29-R	GATAGTGCCGGATACGCTTG
PtrFLA31/Potri.016G088700	PtrFLA31-F	CGGCGATCCTTGCGAAAGCT
	PtrFLA31-R	AGTGTTTGCCACGGTTGCAG
PtrFLA38/ Potri.019G093300	PtrFLA38-F	GGTGGTCAGTTCGTCACATTC
	PtrFLA38-R	CAAACACCTTCTTGGCCAGA
PtrFLA39/ Potri.019G120800	PtrFLA39-F	CCAACGAAACCACTTGTTCT
	PtrFLA39-R	GTACCTAGAGGGTTACTTACA
PtrFLA40/Potri.019G120900	PtrFLA40-F	CCACCCAAGACGAAAACCGC
	PtrFLA40-R	TCTGGTGCAGATGCCACTGG
PtrFLA41/ Potri.019G121100	PtrFLA41-F	GCGTTAAATGATTCAAGCACT
	PtrFLA41-R	GCAACTATACCAACTGACCC
PtrFLA42/Potri.019G121200	PtrFLA42-F	CACCAACGTCACCATAATCC

	PtrFLA42-R	GTCCTTAGGGAGGAGTACCT
PtrFLA43/ Potri.019G121300	PtrFLA43-F	CGGCAGTAGTCGTAGCACCA
	PtrFLA43-R	GCTTGAATCATTTAGTGCGG
PtrFLA44/ Potri.019G122600	PtrFLA44-F	CTACTTCTCAGTTCCAGACC
	PtrFLA44-R	CTCTCTACAGTTACAGCAGG
PtrFLA45/Potri.019G122800	PtrFLA45-F	CTTTGCCCAGTCGCAAGCTG
	PtrFLA45-R	GGCTAGTTGGTTGTCTGTGT
PtrFLA46/ Potri.019G123000	PtrFLA46-F	CTCTAGGTACATGGGCTGGAA
	PtrFLA46-R	GCAACAACTATACCAACTGATC
PtrFLA47/ Potri.019G123100	PtrFLA47-F	ACGTCACCAAAATCCTTGAG
	PtrFLA47-R	TGTACTTAGGGAGGAGCACC
PtrFLA48/ Potri.019G123200	PtrFLA48-F	CGGCAGTAGTCGTAGCACCA
	PtrFLA48-R	GCTTGAATCATTTAGTGCGG
PtrActin2	PtrActin2-F	AACATGGGATTGTTAGCAACTGG
	PtrActin2-R	TCCATCACCAGAATCCAGCACA
Primers used for RT-PCR		
PtrCesA4/Potri.002G257900	PtrCesA4-F	GAGTTAAGGAAGATGGAGAGGTGT
	PtrCesA4-R	TGCACTGAGGACAGGACTGGTTGC
PtrCesA7A/Potri.006G181900	PtrCesA7A-F	TCGCCTTTCTCTCAGATACGAACG
	PtrCesA7A-R	TTACCCGTAACAAGAGGGGGTTCC
PtrCesA7B/Potri.018G103900	PtrCesA7B-F	CCCCTCTAGTCACGGGCAACACAC
	PtrCesA7B-R	AAGGTGCACATTGAAGCACCATCG
PtrCesA8A/Potri.011G069600	PtrCesA8A-F	GTTGGCCTCTGTCTTCTCTCTTGTC
	PtrCesA8A-R	CAATCTATAGAAATGCAGGTTTCAC
PtrCesA8B/Potri.004G059600	PtrCesA8B-F	GTTGGCCTCTGTCTTCTCTCTTGTT

	PtrCesA8B-R	CAATCAATGGAAATGCAGGTCTCCG
PtrPAL4/Potri.010G224100	PtrPAL4-F	ACGTTGGTGTCATGGTGGAG
	PtrPAL4-R	GTGATAGCTTCCAGCATCTC
Ptr4CL3/Potri.001G036900	Ptr4CL3-F	ACGTTGAGCTCACAGCAAGA
	Ptr4CL3-R	ACGCACATGACCTTAACATC
PtrC3H3/Potri.006G033300	PtrC3H3-F	AGTTGGGTGCATCACTTGCA
	PtrC3H3-R	AACACTGTCCAACTCTTCCT
PtrCCoAOMT2/Potri.001G304800	PtrCCoAOMT2-F	AGGAAGGCACCAGGAAGTTG
	PtrCCoAOMT2-R	GTGCGCAACACCAGCTTTCT
PtrF5H2/Potri.007G016400	PtrF5H2-F	GAGAGATGAGGTGGACTCAA
	PtrF5H2-R	GCATCTTCAGAGAAGTTATT
PtrCOMT2/Potri.012G006400	PtrCOMT2-F	GATGCGCCTGTCATGTTAGA
	PtrCOMT2-R	TCCCTTATTGAAGACCTTGT
PtrEXPA1/Potri.001G240900	PtrEXPA1-F	ATGTCATCGTTGTGGATGGC
	PtrEXPA1-R	ACCATCTTGGGTCACTTGCA
PtrEXPA3/Potri.010G167200	PtrEXPA3-F	GGAGGCTGGATTAATGCTCA
	PtrEXPA3-R	AATTGGTGGCGGTGATCACA
PtrEXPA4/Potri.008G088300	PtrEXPA4-F	GGTCTTGCTACGAGATTAGG
	PtrEXPA4-R	ACAACACCAGCACTGTATCG
PtrEXPA5/Potri.009G031800	PtrEXPA5-F	GTCATTGATGTGGAAGGGTG
	PtrEXPA5-R	ACCATTGTGGGTCATCTGCG
PtrEXPA7/Potri.008G057100	PtrEXPA7-F	GTACGGTAACTTGTACAGCC
	PtrEXPA7-R	GGCGATCTTGAGGAACATAG
PtrEXPA16/Potri.006G086100	PtrEXPA16-F	GGCAATCTGTATAGCCAAGG
	PtrEXPA16-R	TCAAAGTGAGGACGAGGTGG

PtrCYCD3;3/Potri.005G141900	PtrCYCD3;3-F	CATTGGCTCTTGACGGGCTT
	PtrCYCD3;3-R	TCAACTGCCTCTCTACGAAC
