# Supplementary material for: Cas9/gRNA-Mediated Mutations in PtrFLA40 and PtrFLA45 Reveal Redundant Roles in Modulating Wood Cell Size and SCW Synthesis in Poplar
Source: Int J Mol Sci. 2022 Dec 27;24(1):427. doi: 10.3390/ijms24010427 (PMC9820481; doi:10.3390/ijms24010427)
Supplement: Supplementary file 1 [file ijms-24-00427-s001.zip › Table S4.pdf]

Table S4. The Cas9/gRNA-targeted mutations in *PtrFLA40* and *PtrFLA45.*

Gene: Potri.019G120900 (*PtrFLA40*)

Vector: Cas9/gRNA-*PtrFLA40*
gRNA: g*PtrFLA40*-T1 (**CCC**AAGACGAAAACCGCTTATTC); g*PtrFLA40*-T2 (GATATATGTAGGGTTGTGTC**CGG**)
Target sequences: *PtrFLA40*-T1, **CCC**AAGACGAAAACCGCTTATTC; *PtrFLA40*-T2, GATATATGTAGGGTTGTGTC**CGG**

**Line (#)**	*PtrFLA40*-T1<br>**CCC**AAGACGAAAACCGCTTATTC	**Mutation types**<br>(Number of plasmids containing the cloned PCR products)		*PtrFLA40*-T2<br>GATATATGTAGGGTTGTGTC**CGG**	**Mutation types**<br>(Number of plasmids containing the cloned PCR products)	
ptrfla40-1#	**CCC**AAGACGAAA-CCGCTTATTC<br>**CCC**AAGA-GAAAACCGCTTATTC<br>**CCC**AAGACGAAAACCGCTTATTC	-1 (×8)<br>-1 (×7)<br>0 (×5)	chimera	GATATATGTAGGGTTGTGTC**CGG**	0 (×20)	WT
ptrfla40-2#	**CCC**AAGACGAAA-CCGCTTATTC	-1 (×20)	homozygous	GATATATGTAGGGTTGTGTC**CGG**	0 (×20)	WT
ptrfla40-3#	**CCC**AAGA(A)CGAAAACCGCTTATTC	+1 (×20)	homozygous	GATATATGTAGGGTTGTGTC**CGG**	0 (×20)	WT
ptrfla40-4#	**CCC**AAGACGAAA-CCGCTTATTC<br>**CCC**AAG(T)ACGAAAACCGCTTATTC<br>**CCC**AAGACGAAAACCGCTTATTC	-1 (×12)<br>+1 (×5)<br>0 (×3)	chimera	GATATATGTAGGGTTGTGTC**CGG**	0 (×20)	WT
ptrfla40-5#	**CCC**AAG(T)ACGAAAACCGCTTATTC<br>**CCC**AAGACGAAAACCGCTTATTC	+1 (×13)<br>0 (×7)	heterozygous	GATATATGTAGGGTTGTGTC**CGG**	0 (×20)	WT
ptrfla40-6#	**CCC**AAGA(A)CGAAAACCGCTTATTC<br>**CCC**AAGACGAAA-CCGCTTATTC	+1 (×11)<br>-1 (×9)	biallelic	GATATATGTAGGGTTGTGTC**CGG**	0 (×20)	WT

Gene: Potri.019G122800 (*PtrFLA45*)

Vector: Cas9/gRNA-*PtrFLA45*
gRNA: g*PtrFLA45-T1* (**CCC**TGGTTTAACCATCTTTGCAC); g*PtrFLA45-T2* (GTCACAAAGTTATACAAACT**CGG**)
Target sequences: *PtrFLA45-T1*, **CCC**TGGTTTAACCATCTTTGCAC; *PtAP66*-4, GTCACAAAGTTATACAAACT**CGG**

**Line (#)**	*PtrFLA45*-T1<br>**CCC**TGGTTTAACCATCTTTGCAC	**Mutation types**<br>(Number of plasmids containing the cloned PCR products)		*PtrFLA45*-T2<br>GTCACAAAGTTATACAAACT**CGG**	**Mutation types**<br>(Number of plasmids containing the cloned PCR products)	
ptrfla45-1#	**CCC**TGGT-----------------------------------......	-178 (×20)	homozygous	......-------------------------------- ACT**CGG**	-178 (×20)	homozygous

ptrfla45-2#	**CCC**T-----AACCATCTTTGCAC	-5 (×20)	homozygous	GTCACAAAGTTATACAA-CT**CGG**	-1 (×20)	homozygous
ptrfla45-3#	CCCTGG-----CCATCTTTGCAC<br>**CCC**TGGTTTAACCATCTTTGCAC	-5 (×13)<br>0 (×7)	heterozygous	GTCACAAAGTTATACAA--T**CGG**<br>GTCACAAAGTTATACAAACT**CGG**	-2 (×9)<br>0 (×11)	heterozygous
ptrfla45-4#	**CCC**TGGT----------------------------------......<br>**CCC**TGGTTTAACCATCTTTGCAC	-178 (×14)<br>0 (×6)	heterozygous	......------------------------------- ACT**CG**G<br>GTCACAAAGTTATACAAACT**CGG**	-178 (×14)<br>0 (×6)	heterozygous
ptrfla45-5#	**CCC**TGG-----------------------------------......<br>**CCC**TGGTTTAACCATCTTTGCAC	-179 (×11)<br>0 (×9)	heterozygous	......--------------------------------ACT**CGG**<br>GTCACAAAGTTATACAAACT**CGG**	-179 (×11)<br>0 (×9)	heterozygous
ptrfla45-6#	**CCC**TGGTTT----------GCAC	-10 (×20)	homozygous	GTCACAAAGTTATACAAACT**CGG**	0 (×20)	homozygous
ptrfla45-7#	**CCC**TGGT----------------------------------......<br>**CCC**TGG-----------------------------------......<br>**CCC**TGGTTTAACCATCTTTGCAC	-178 (×9)<br>-179 (×7)<br>0 (×4)	chimera	......------------------------------- ACT**CGG**<br>......--------------------------------ACT**CGG**<br>GTCACAAAGTTATACAAACT**CGG**	-178 (×9)<br>-179 (×7)<br>0 (×4)	chimera

Vector: Cas9/gRNA-*PtrFLA40/45*
gRNA: g*PtrFLA40/45-T1* (**CCA**TTGTCCCAATACCTTTGCCC); g*PtrFLA40/45-T2* (GCGCTAAATGACTCAAGCCC**TGG**)
Target sequences: *PtrFLA40-T1*, **CCA**TTGTCCCAATACCTTTGCCC; *PtrFLA40-T2*, GCGCTAAATGACTCAAGCAC**TGG**
PtrFLA45-T1, **CCA**TTGTCCCAATACCTTTGCCC; *PtrFLA45-T2*, GCGCTAAATGACTCAAGCCC**TGG**

**Line (#)**	*PtrFLA40/45*-T1<br>*PtrFLA40*, **CCA**TTGTCCCAATACCTTTGCCC<br>*PtrFLA45*, **CCA**TTGTCCCAATACCTTTGCCC	**Mutation types** (Number of plasmids containing the cloned PCR products)		*PtrFLA40/45*-T2<br>*PtrFLA40*,GCGCTAAATGACTCAAGCAC**TGG**<br>*PtrFLA45*,GCGCTAAATGACTCAAGCCC**TGG**	**Mutation types** (Number of plasmids containing the cloned PCR products)	
ptrfla40/45-1#-fla40	**CCA**TT(A)GTCCCAATACCTTTGCCC<br>------------------TGCCC	+1 (×15)<br>-18 (×5)	biallelic	GCGCTAAATGACTCAAGCAC**TGG**	0 (×20)	WT
-fla45	**CCA**---------------------------------------......	-222 (×20)	homozygous	......-------------------------------------CCCTGG	-222 (×20)	homozygous
ptrfla40/45-2#-fla40	**CCA**T-GTCCCAATACCTTTGCCC	-1 (×20)	homozygous	GCGCTAAATGACTCAAGCAC**TGG**	0 (×20)	WT
-fla45	**CCA**T-GTCCCAATACCTTTGCCC	-1 (×20)	homozygous	GCGCTAAATGACTCAAG(A)CCC**TGG**	+1 (×20)	homozygous
ptrfla40/45-3#-fla40	**CCA**T-GTCCCAATACCTTTGCCC<br>**CCA**TTGTCCCAATACCTTTGCCC	-1 (×14)<br>0 (×6)	heterozygous	GCGCTAAATGACTCAAGCAC**TGG**	0 (×20)	WT
-fla45	**CCA**T-GTCCCAATACCTTTGCCC<br>-------TACCAATACCTTTGCCC	-1 (×12)<br>-7 (×8)	biallelic	--------------------**TGG**<br>GCGCTAAATGACTCAAGCCC**TGG**	-20 (×12)<br>0 (×8)	heterozygous
ptrfla40/45-4#-fla40	**C**-----TACCAATACCTTTGCCC<br>**CCA**--GTCCCAATACCTTTGCCC<br>**CCA**TTGTACCAATACCTTTGCCC	-5 (×8)<br>-2 (×3)<br>0 (×9)	chimera	GCGCTAAATGACTCAAGCAC**TGG**	0 (×20)	WT
-fla45	**CCA**T-GTCCCAATACCTTTGCCC	-1 (×20)	homozygous	--------------------**TGG**	-20 (×20)	homozygous

ptrfla40/45-5#-fla40	**C**-----TCCCAATACCTTTGCCC	-5 (×20)	homozygous	GCGCTAAATGACTCAAGCAC**TGG**	0 (×20)	WT
-fla45	**C**--TTGTCCCAATACCTTTGCCC	-2 (×20)	homozygous	GCGCTAAATGAC-----CCC**TGG**	-5 (×20)	homozygous
ptrfla40/45-6#-fla40	**CCA**T-GTCCCAATACCTTTGCCC<br>**CCA**TTGTCCCAATACCTTTGCCC	-1 (×11)<br>0 (×9)	heterozygous	GCGCTAAATGACTCAAGCAC**TGG**	0 (×20)	WT
-fla45	**CCA**T-GTCCCAATACCTTTGCCC<br>-------TACCAATACCTTTGCCC<br>**CCA**TTGTACCAATACCTTTGCCC	-1 (×4)<br>-7 (×7)<br>0 (×9)	chimera	GCGCTAAATGACTCAAG(G)CCC**TGG**<br>GCGCTAAATGACTCAAGCCC**TGG**	+1 (×8)<br>0 (×12)	heterozygous
ptrfla40/45-7#-fla40	......--......	-254 (×20)	homozygous	GCGCTAAATGACTCAAGCAC**TGG**	0 (×20)	homozygous
-fla45	**C**--TTGTCCCAATACCTTTGCCC<br>**CCA**T-GTCCCAATACCTTTGCCC<br>-------TACCAATACCTTTGCCC<br>......--......	-2 (×2)<br>-1 (×5)<br>-7 (×7)<br>-254 (×6)	chimera	GCGCTAAATGAC-----CCC**TGG**<br>--------------------**TGG**<br>GCGCTAAATGACTCAAGCCC**TGG**<br>......--......	-5 (×2)<br>-20 (×5)<br>0 (×7)<br>-254(×6)	chimera
ptrfla40/45-8#-fla40	......--......	-254 (×20)	homozygous	GCGCTAAATGACTCAAGCAC**TGG**	0 (×20)	homozygous
-fla45	**CCA**T-GTCCCAATACCTTTGCCC<br>-------TACCAATACCTTTGCCC	-1 (×15)<br>-7 (×5)	biallelic	......-- C**TGG**<br>--------------------**TGG**<br>GCGCTAAATGACTCAAGCCC**TGG**	-191 (×1)<br>-20 (×14)<br>0 (×5)	chimera
ptrfla40/45-9#-fla40	**CCA**--GTCCCAATACCTTTGCCC	-2 (×20)	homozygous	GCGCTAAATGACTCAAGCAC**TGG**	0 (×20)	WT
-fla45	**CCA**T-GTCCCAATACCTTTGCCC<br>-------TACCAATACCTTTGCCC	-1 (×18)<br>-7 (×2)	biallelic	--------------------**TGG**<br>GCGCTAAATGACTCAAGCCC**TGG**	-20 (×18)<br>0 (×2)	heterozygous
